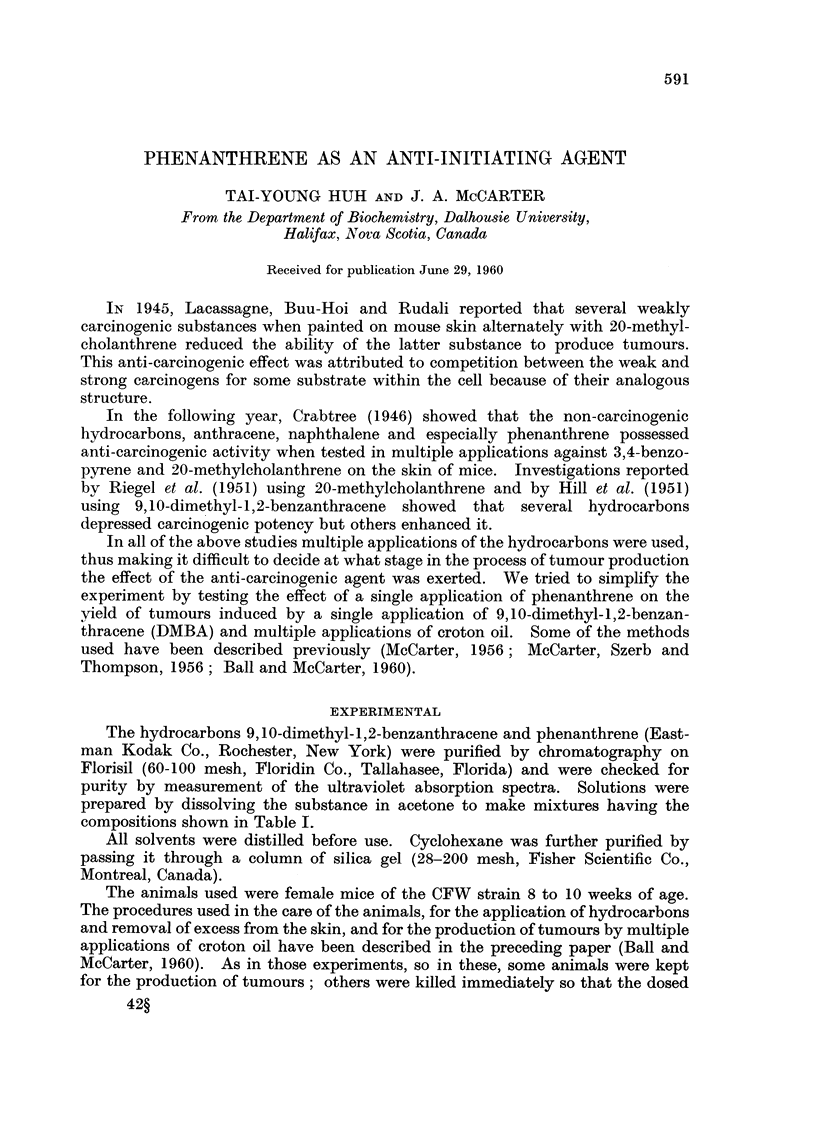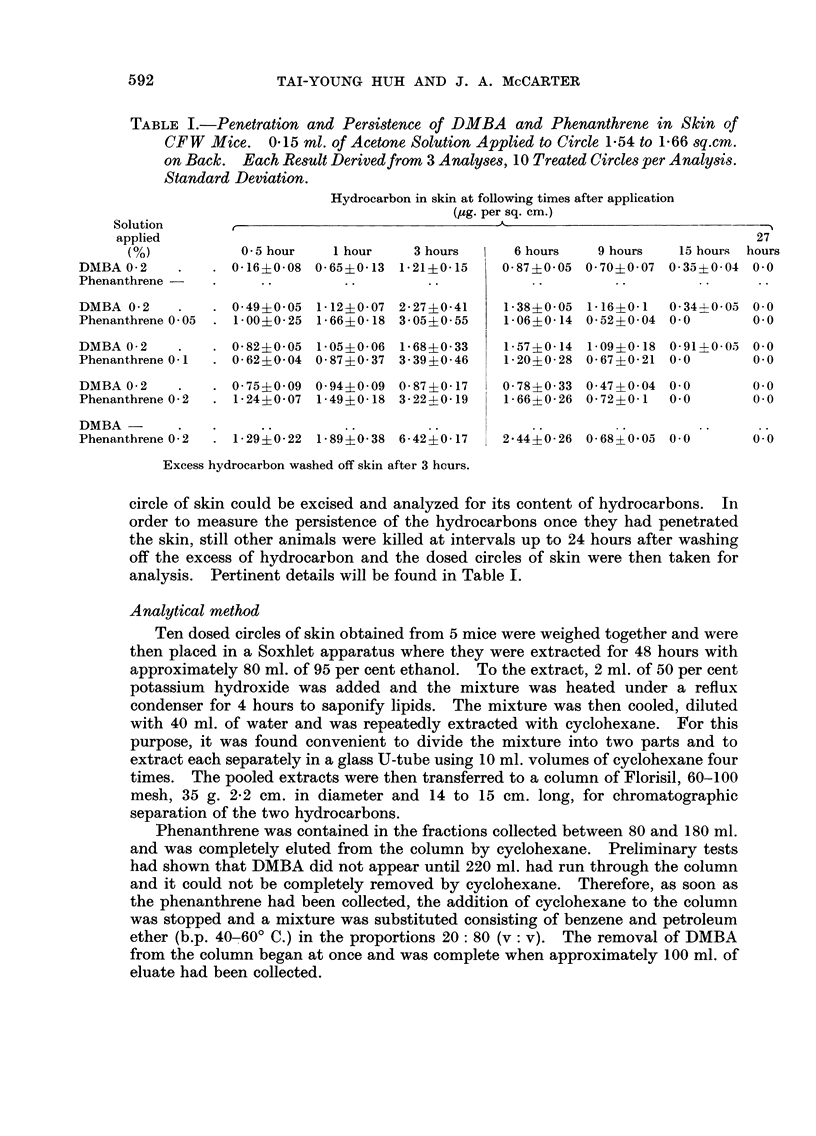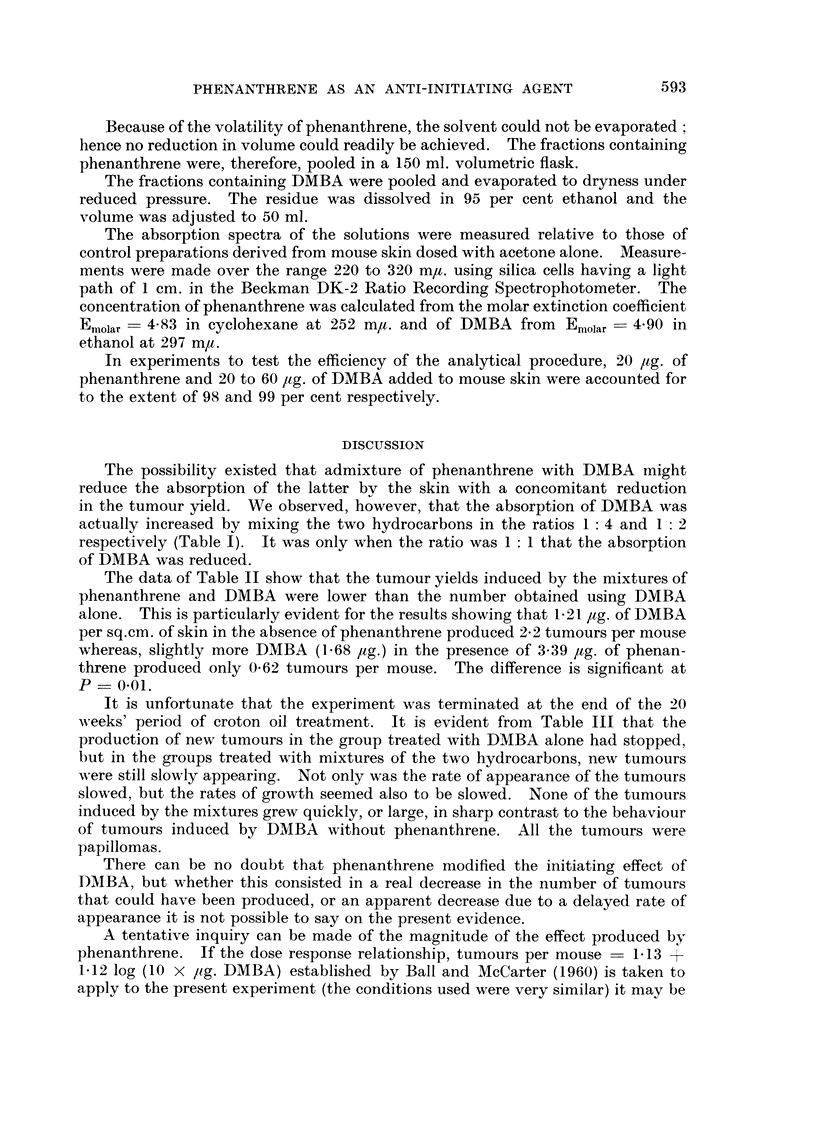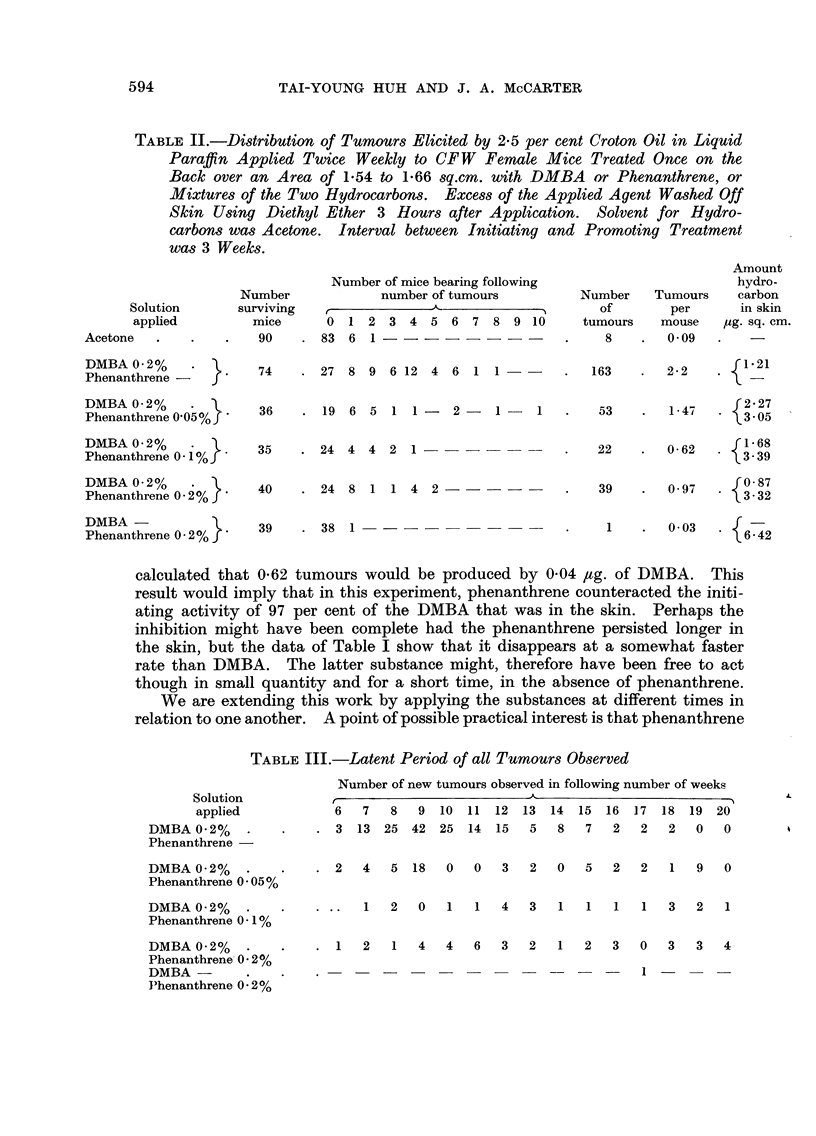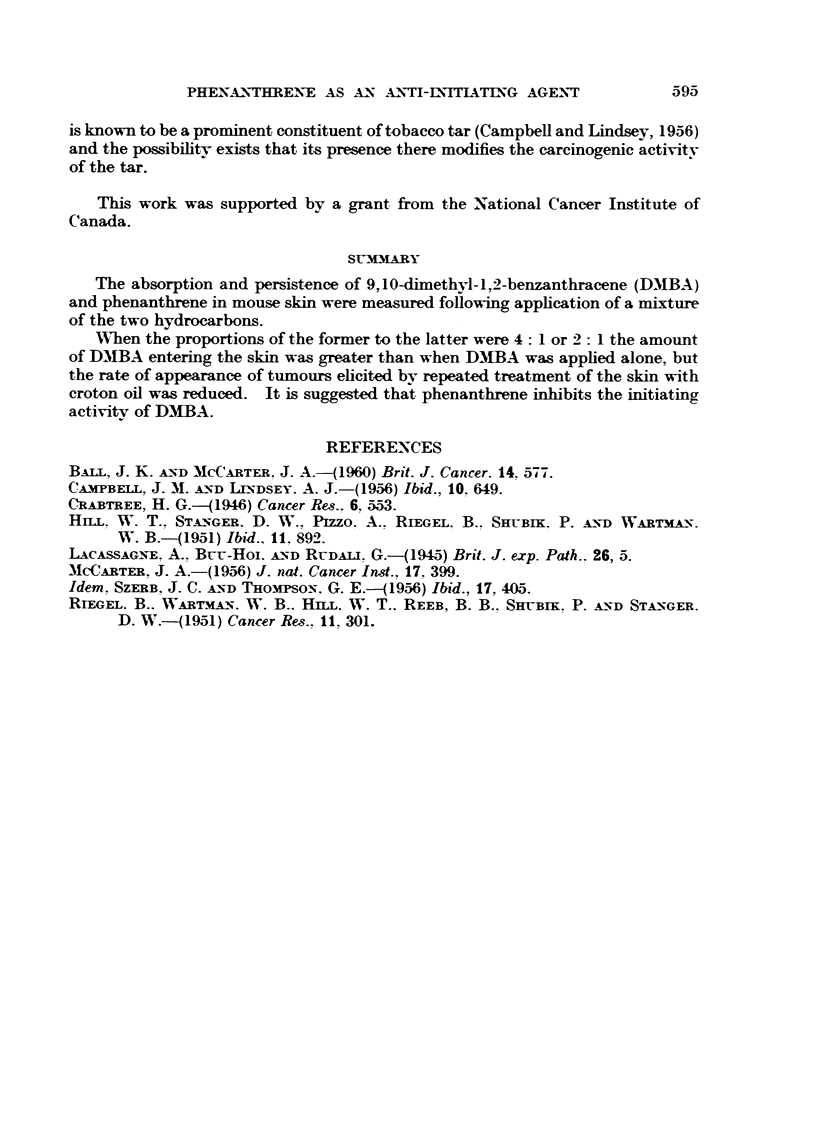# Phenanthrene as an Anti-initiating Agent

**DOI:** 10.1038/bjc.1960.64

**Published:** 1960-09

**Authors:** Tai-Young Huh, J. A. McCarter


### PHENANTHRENE AS AN ANTI-INITIATING AGENT

TAI-YOUNG HUH AND J. A. McCARTER
*From the Department of Biochemistry, Dalhousie University, Halifax, Nova Scotia, Canada*

Received for publication June 29, 1960

IN 1945, Lacassagne, Buu-Hoi and Rudali reported that several weakly carcinogenic substances when painted on mouse skin alternately with 20-methylcholanthrene reduced the ability of the latter substance to produce tumours. This anti-carcinogenic effect was attributed to competition between the weak and strong carcinogens for some substrate within the cell because of their analogous structure.

In the following year, Crabtree (1946) showed that the non-carcinogenic hydrocarbons, anthracene, naphthalene and especially phenanthrene possessed anti-carcinogenic activity when tested in multiple applications against 3,4-benzopyrene and 20-methylcholanthrene on the skin of mice. Investigations reported by Riegel *et al.* (1951) using 20-methylcholanthrene and by Hill *et al.* (1951) using 9,10-dimethyl-1,2-benzanthracene showed that several hydrocarbons depressed carcinogenic potency but others enhanced it.

In all of the above studies multiple applications of the hydrocarbons were used, thus making it difficult to decide at what stage in the process of tumour production the effect of the anti-carcinogenic agent was exerted. We tried to simplify the experiment by testing the effect of a single application of phenanthrene on the yield of tumours induced by a single application of 9,10-dimethyl-1,2-benzanthracene (DMBA) and multiple applications of croton oil. Some of the methods used have been described previously (McCarter, 1956; McCarter, Szerb and Thompson, 1956; Ball and McCarter, 1960).

### EXPERIMENTAL

The hydrocarbons 9,10-dimethyl-1,2-benzanthracene and phenanthrene (Eastman Kodak Co., Rochester, New York) were purified by chromatography on Florisil (60-100 mesh, Floridin Co., Tallahasee, Florida) and were checked for purity by measurement of the ultraviolet absorption spectra. Solutions were prepared by dissolving the substance in acetone to make mixtures having the compositions shown in Table I.

All solvents were distilled before use. Cyclohexane was further purified by passing it through a column of silica gel (28–200 mesh, Fisher Scientific Co., Montreal, Canada).

The animals used were female mice of the CFW strain 8 to 10 weeks of age. The procedures used in the care of the animals, for the application of hydrocarbons and removal of excess from the skin, and for the production of tumours by multiple applications of croton oil have been described in the preceding paper (Ball and McCarter, 1960). As in those experiments, so in these, some animals were kept for the production of tumours; others were killed immediately so that the dosed

TABLE I.—*Penetration and Persistence of DMBA and Phenanthrene in Skin of CFW Mice. 0·15 ml. of Acetone Solution Applied to Circle 1·54 to 1·66 sq.cm. on Back. Each Result Derived from 3 Analyses, 10 Treated Circles per Analysis. Standard Deviation.*

| Solution applied (%) | Hydrocarbon in skin at following times after application ($\mu$g. per sq. cm.) 0·5 hour | 1 hour | 3 hours | 6 hours | 9 hours | 15 hours | 27 hours |
|---|---|---|---|---|---|---|---|
| DMBA 0·2 | 0·16±0·08 | 0·65±0·13 | 1·21±0·15 | 0·87±0·05 | 0·70±0·07 | 0·35±0·04 | 0·0 |
| Phenanthrene — | .. | .. | .. | .. | .. | .. | .. |
| DMBA 0·2 | 0·49±0·05 | 1·12±0·07 | 2·27±0·41 | 1·38±0·05 | 1·16±0·1 | 0·34±0·05 | 0·0 |
| Phenanthrene 0·05 | 1·00±0·25 | 1·66±0·18 | 3·05±0·55 | 1·06±0·14 | 0·52±0·04 | 0·0 | 0·0 |
| DMBA 0·2 | 0·82±0·05 | 1·05±0·06 | 1·68±0·33 | 1·57±0·14 | 1·09±0·18 | 0·91±0·05 | 0·0 |
| Phenanthrene 0·1 | 0·62±0·04 | 0·87±0·37 | 3·39±0·46 | 1·20±0·28 | 0·67±0·21 | 0·0 | 0·0 |
| DMBA 0·2 | 0·75±0·09 | 0·94±0·09 | 0·87±0·17 | 0·78±0·33 | 0·47±0·04 | 0·0 | 0·0 |
| Phenanthrene 0·2 | 1·24±0·07 | 1·49±0·18 | 3·22±0·19 | 1·66±0·26 | 0·72±0·1 | 0·0 | 0·0 |
| DMBA — | .. | .. | .. | .. | .. | .. | .. |
| Phenanthrene 0·2 | 1·29±0·22 | 1·89±0·38 | 6·42±0·17 | 2·44±0·26 | 0·68±0·05 | 0·0 | 0·0 |

Excess hydrocarbon washed off skin after 3 hours.

circle of skin could be excised and analyzed for its content of hydrocarbons. In order to measure the persistence of the hydrocarbons once they had penetrated the skin, still other animals were killed at intervals up to 24 hours after washing off the excess of hydrocarbon and the dosed circles of skin were then taken for analysis. Pertinent details will be found in Table I.

*Analytical method*

Ten dosed circles of skin obtained from 5 mice were weighed together and were then placed in a Soxhlet apparatus where they were extracted for 48 hours with approximately 80 ml. of 95 per cent ethanol. To the extract, 2 ml. of 50 per cent potassium hydroxide was added and the mixture was heated under a reflux condenser for 4 hours to saponify lipids. The mixture was then cooled, diluted with 40 ml. of water and was repeatedly extracted with cyclohexane. For this purpose, it was found convenient to divide the mixture into two parts and to extract each separately in a glass U-tube using 10 ml. volumes of cyclohexane four times. The pooled extracts were then transferred to a column of Florisil, 60–100 mesh, 35 g. 2·2 cm. in diameter and 14 to 15 cm. long, for chromatographic separation of the two hydrocarbons.

Phenanthrene was contained in the fractions collected between 80 and 180 ml. and was completely eluted from the column by cyclohexane. Preliminary tests had shown that DMBA did not appear until 220 ml. had run through the column and it could not be completely removed by cyclohexane. Therefore, as soon as the phenanthrene had been collected, the addition of cyclohexane to the column was stopped and a mixture was substituted consisting of benzene and petroleum ether (b.p. 40–60° C.) in the proportions 20 : 80 (v : v). The removal of DMBA from the column began at once and was complete when approximately 100 ml. of eluate had been collected.

Because of the volatility of phenanthrene, the solvent could not be evaporated ; hence no reduction in volume could readily be achieved. The fractions containing phenanthrene were, therefore, pooled in a 150 ml. volumetric flask.

The fractions containing DMBA were pooled and evaporated to dryness under reduced pressure. The residue was dissolved in 95 per cent ethanol and the volume was adjusted to 50 ml.

The absorption spectra of the solutions were measured relative to those of control preparations derived from mouse skin dosed with acetone alone. Measurements were made over the range 220 to 320 m$\mu$. using silica cells having a light path of 1 cm. in the Beckman DK-2 Ratio Recording Spectrophotometer. The concentration of phenanthrene was calculated from the molar extinction coefficient $E_{molar} = 4{\cdot}83$ in cyclohexane at 252 m$\mu$. and of DMBA from $E_{molar} = 4{\cdot}90$ in ethanol at 297 m$\mu$.

In experiments to test the efficiency of the analytical procedure, 20 $\mu$g. of phenanthrene and 20 to 60 $\mu$g. of DMBA added to mouse skin were accounted for to the extent of 98 and 99 per cent respectively.

### DISCUSSION

The possibility existed that admixture of phenanthrene with DMBA might reduce the absorption of the latter by the skin with a concomitant reduction in the tumour yield. We observed, however, that the absorption of DMBA was actually increased by mixing the two hydrocarbons in the ratios 1 : 4 and 1 : 2 respectively (Table I). It was only when the ratio was 1 : 1 that the absorption of DMBA was reduced.

The data of Table II show that the tumour yields induced by the mixtures of phenanthrene and DMBA were lower than the number obtained using DMBA alone. This is particularly evident for the results showing that 1·21 $\mu$g. of DMBA per sq.cm. of skin in the absence of phenanthrene produced 2·2 tumours per mouse whereas, slightly more DMBA (1·68 $\mu$g.) in the presence of 3·39 $\mu$g. of phenanthrene produced only 0·62 tumours per mouse. The difference is significant at $P = 0{\cdot}01$.

It is unfortunate that the experiment was terminated at the end of the 20 weeks' period of croton oil treatment. It is evident from Table III that the production of new tumours in the group treated with DMBA alone had stopped, but in the groups treated with mixtures of the two hydrocarbons, new tumours were still slowly appearing. Not only was the rate of appearance of the tumours slowed, but the rates of growth seemed also to be slowed. None of the tumours induced by the mixtures grew quickly, or large, in sharp contrast to the behaviour of tumours induced by DMBA without phenanthrene. All the tumours were papillomas.

There can be no doubt that phenanthrene modified the initiating effect of DMBA, but whether this consisted in a real decrease in the number of tumours that could have been produced, or an apparent decrease due to a delayed rate of appearance it is not possible to say on the present evidence.

A tentative inquiry can be made of the magnitude of the effect produced by phenanthrene. If the dose response relationship, tumours per mouse $= 1{\cdot}13 + 1{\cdot}12 \log (10 \times \mu\text{g. DMBA})$ established by Ball and McCarter (1960) is taken to apply to the present experiment (the conditions used were very similar) it may be

TABLE II.—*Distribution of Tumours Elicited by 2·5 per cent Croton Oil in Liquid Paraffin Applied Twice Weekly to CFW Female Mice Treated Once on the Back over an Area of 1·54 to 1·66 sq.cm. with DMBA or Phenanthrene, or Mixtures of the Two Hydrocarbons. Excess of the Applied Agent Washed Off Skin Using Diethyl Ether 3 Hours after Application. Solvent for Hydrocarbons was Acetone. Interval between Initiating and Promoting Treatment was 3 Weeks.*

| Solution applied | Number surviving mice | Number of mice bearing following number of tumours: 0 | 1 | 2 | 3 | 4 | 5 | 6 | 7 | 8 | 9 | 10 | Number of tumours | Tumours per mouse | Amount hydrocarbon in skin μg. sq. cm. |
|---|---|---|---|---|---|---|---|---|---|---|---|---|---|---|---|
| Acetone | 90 | 83 | 6 | 1 | — | — | — | — | — | — | — | — | 8 | 0·09 | — |
| DMBA 0·2%<br>Phenanthrene — | 74 | 27 | 8 | 9 | 6 | 12 | 4 | 6 | 1 | 1 | — | — | 163 | 2·2 | 1·21<br>— |
| DMBA 0·2%<br>Phenanthrene 0·05% | 36 | 19 | 6 | 5 | 1 | 1 | — | 2 | — | 1 | — | 1 | 53 | 1·47 | 2·27<br>3·05 |
| DMBA 0·2%<br>Phenanthrene 0·1% | 35 | 24 | 4 | 4 | 2 | 1 | — | — | — | — | — | — | 22 | 0·62 | 1·68<br>3·39 |
| DMBA 0·2%<br>Phenanthrene 0·2% | 40 | 24 | 8 | 1 | 1 | 4 | 2 | — | — | — | — | — | 39 | 0·97 | 0·87<br>3·32 |
| DMBA —<br>Phenanthrene 0·2% | 39 | 38 | 1 | — | — | — | — | — | — | — | — | — | 1 | 0·03 | —<br>6·42 |

calculated that 0·62 tumours would be produced by 0·04 μg. of DMBA. This result would imply that in this experiment, phenanthrene counteracted the initiating activity of 97 per cent of the DMBA that was in the skin. Perhaps the inhibition might have been complete had the phenanthrene persisted longer in the skin, but the data of Table I show that it disappears at a somewhat faster rate than DMBA. The latter substance might, therefore have been free to act though in small quantity and for a short time, in the absence of phenanthrene.

We are extending this work by applying the substances at different times in relation to one another. A point of possible practical interest is that phenanthrene

TABLE III.—*Latent Period of all Tumours Observed*

| Solution applied | Number of new tumours observed in following number of weeks: 6 | 7 | 8 | 9 | 10 | 11 | 12 | 13 | 14 | 15 | 16 | 17 | 18 | 19 | 20 |
|---|---|---|---|---|---|---|---|---|---|---|---|---|---|---|---|
| DMBA 0·2%<br>Phenanthrene — | 3 | 13 | 25 | 42 | 25 | 14 | 15 | 5 | 8 | 7 | 2 | 2 | 2 | 0 | 0 |
| DMBA 0·2%<br>Phenanthrene 0·05% | 2 | 4 | 5 | 18 | 0 | 0 | 3 | 2 | 0 | 5 | 2 | 2 | 1 | 9 | 0 |
| DMBA 0·2%<br>Phenanthrene 0·1% | .. | 1 | 2 | 0 | 1 | 1 | 4 | 3 | 1 | 1 | 1 | 1 | 3 | 2 | 1 |
| DMBA 0·2%<br>Phenanthrene 0·2% | 1 | 2 | 1 | 4 | 4 | 6 | 3 | 2 | 1 | 2 | 3 | 0 | 3 | 3 | 4 |
| DMBA —<br>Phenanthrene 0·2% | — | — | — | — | — | — | — | — | — | — | — | 1 | — | — | — |

is known to be a prominent constituent of tobacco tar (Campbell and Lindsey, 1956) and the possibility exists that its presence there modifies the carcinogenic activity of the tar.

This work was supported by a grant from the National Cancer Institute of Canada.

### SUMMARY

The absorption and persistence of 9,10-dimethyl-1,2-benzanthracene (DMBA) and phenanthrene in mouse skin were measured following application of a mixture of the two hydrocarbons.

When the proportions of the former to the latter were 4 : 1 or 2 : 1 the amount of DMBA entering the skin was greater than when DMBA was applied alone, but the rate of appearance of tumours elicited by repeated treatment of the skin with croton oil was reduced. It is suggested that phenanthrene inhibits the initiating activity of DMBA.

### REFERENCES

BALL, J. K. AND McCARTER, J. A.—(1960) *Brit. J. Cancer*, **14**, 577.

CAMPBELL, J. M. AND LINDSEY, A. J.—(1956) *Ibid.*, **10**, 649.

CRABTREE, H. G.—(1946) *Cancer Res.*, **6**, 553.

HILL, W. T., STANGER, D. W., PIZZO, A., RIEGEL, B., SHUBIK, P. AND WARTMAN, W. B.—(1951) *Ibid.*, **11**, 892.

LACASSAGNE, A., BUU-HOI, AND RUDALI, G.—(1945) *Brit. J. exp. Path.*, **26**, 5.

McCARTER, J. A.—(1956) *J. nat. Cancer Inst.*, **17**, 399.

*Idem*, SZERB, J. C. AND THOMPSON, G. E.—(1956) *Ibid.*, **17**, 405.